# Atypical hemolytic uremic syndrome: a case report

**DOI:** 10.1186/s13256-019-2334-y

**Published:** 2020-01-13

**Authors:** B. M. D. B. Basnayake, A. W. M. Wazil, N. Nanayakkara, S. M. D. K. Samarakoon, E. M. S. K. Senavirathne, B. U. E. W. D. R. Thangarajah, N. Karunasena, R. M. B. S. S. Mahanama

**Affiliations:** 1 Department of Nephrology and Renal Transplant, Teaching Hospital Kandy, Kandy, Sri Lanka; 2 Department of Transfusion Medicine, Teaching Hospital Kandy, Kandy, Sri Lanka

**Keywords:** Atypical hemolytic uremic syndrome, Thrombotic microangiopathy, Sri Lanka

## Abstract

**Background:**

Thrombotic microangiopathy is a pathological condition comprised of microvascular thrombosis involving any organ of the body leading to thrombocytopenia, Coombs-negative hemolytic anemia, and end-organ damage. The most common forms of thrombotic microangiopathies are Shiga toxin-producing *Escherichia coli*-mediated hemolytic uremic syndrome, thrombotic thrombocytopenic purpura, and atypical hemolytic uremic syndrome. The atypical hemolytic uremic syndrome occurs due to genetic and acquired mutations in complement regulatory factors and to complement activation factors in the immune system, mainly the alternative pathway. Clinical manifestations and outcomes differ with the prevalent mutations of the patient. Currently, available treatment modalities are therapeutic plasma exchange and a monoclonal antibody against C5, eculizumab. We report a case of a Sri Lankan girl diagnosed with atypical hemolytic uremic syndrome complicated with septicemia, hemolytic anemia, acute kidney injury, pulmonary hemorrhage with respiratory failure, and hypertension who had a complete remission following long-term (30 months) therapeutic plasma exchange.

**Case presentation:**

A 15-year-old Sri Lankan girl was transferred from a local hospital with the features of septicemia and acute kidney injury for specialized management. She had high blood pressure (180/100 mmHg) on admission. She underwent appendicectomy based on suspicion of acute appendicitis as the cause of sepsis. Following surgery, her condition deteriorated, and intensive care unit management was warranted because she developed pulmonary hemorrhages and respiratory failure requiring mechanical ventilation and renal replacement therapy in the form of hemodialysis. Her blood investigations showed microangiopathic hemolytic anemia, thrombocytopenia, elevated lactate dehydrogenase, and reduced human complement C3 levels, together with a normal coagulation profile. She was diagnosed with atypical hemolytic uremic syndrome and was initiated on therapeutic plasma exchange and other supportive therapy, including corticosteroids. Following a lengthy course of plasma exchange, complete recovery was achieved.

**Conclusion:**

The atypical hemolytic uremic syndrome is a rare disease entity requiring a high index of suspicion to diagnose. It is a diagnosis of exclusion. Early diagnosis with prompt treatment will render a better outcome. The atypical hemolytic uremic syndrome needs to be considered in all patients with thrombotic microangiopathy.

## Background

Thrombotic microangiopathy (TMA) is characterized by thrombocytopenia and Coombs test-negative hemolytic anemia with end-organ damage. Any organ of the body can be involved, commonly the kidney, central nervous system, gastrointestinal tract, lungs, and heart [1]. TMA is a pathological condition resulting in thrombosis of capillaries and arterioles. It is due to endothelial injury that leads to the activation of platelets causing platelet aggregation and thrombus formation. Due to the consumption of platelets, thrombocytopenia ensues. Small vessel occlusion leads to organ hypoxia and ischemic organ damage [1, 2]. According to recent updates, TMA is classified into three major categories. These include Shiga toxin-producing *Escherichia coli* (STEC) hemolytic uremic syndrome (HUS), thrombotic thrombocytopenic purpura (TTP), and atypical hemolytic uremic syndrome (aHUS), which is further classified according to the etiology. Etiology-based nomenclature, such as pregnancy-induced aHUS, post-transplant aHUS, drug-induced, infection-induced aHUS, and autoimmune disease-associated aHUS, is also widely used currently [3].

Even though the clinical manifestations of TMA are similar, pathophysiologies differ from each other. TTP occurs due to severe deficiency of ADAMTS-13 (a disintegrin and metalloproteinase with thrombospondin type 1 motif, member 13). ADAMTS-13 is a specific cleaving protease of von Willebrand factor (vWF). Absent cleavage of ultralarge vWF multimers due to lack of ADAMTS-13 activity will promote thrombus formation in small vessels [4, 5]. In STEC-HUS, Shiga toxin exerts direct toxicity to cells by binding to the globotriaosylceramide receptors on the target cell surface and escorting to cytotoxicity. Cytotoxicity includes protein synthesis and apoptosis. Shiga toxin also increases the secretion of abnormally sized vWF multimers from endothelial cells [4, 6]. aHUS is due to the uncontrolled complement activation [7]. We report a case of a Sri Lankan patient diagnosed with aHUS who was treated successfully with plasma exchange therapy. To our knowledge, there is only a handful of case reports on aHUS in Sri Lanka, and this is the only reported case with complete recovery following a lengthy course of plasma exchange in Sri Lanka.

## Case presentation

A 15-year-old, previously healthy Sri Lankan girl with no significant medical history or family history of hypertension or diabetes mellitus was admitted to a peripheral hospital with fever and skin rash of 2 weeks’ duration that was treated as chickenpox. The diagnosis of chickenpox was confirmed by a dermatologist. She developed severe lower abdominal pain. She was oliguric and tachycardic (pulse rate of 102 beats per minute), and her blood pressure was elevated to 180/100 mm Hg. Her temperature was 38.1 °C. The result of her neurological examination was normal. Her serum creatinine was 210 μmol/L (normal range 80–130 μmol/L), and she had active urinary sediments (urine full report, red cells 100–150 with 50% dysmorphic red cells). She was clinically diagnosed with acute appendicitis, confirmed with ultrasound findings and complicated with sepsis-induced acute kidney injury. She was initiated on intravenous cefuroxime 750 mg 8-hourly and intravenous metronidazole 500 mg 8-hourly and was transferred to the nephrology unit of Teaching Hospital Kandy for specialized care.

She was referred to the surgical unit, where an appendectomy was performed while she was under general anesthesia. Her appendix was inflamed, but it was not perforated. Both intravenous cefuroxime 750 mg 8-hourly and intravenous metronidazole 500 mg 8-hourly were continued. In preoperative assessment, her blood pressure was 150/94 mmHg, and her serum creatinine level was high (226 μmol/L) with hyperkalemia (5.8 mmol/L), which was corrected with a potassium-lowering regimen prior to induction of anesthesia. During the surgery, her blood pressure was under control, and her recovery was also uneventful. Later, the diagnosis of appendicitis was confirmed with the histological findings.

On postsurgery day 1, she was anuric and severely acidotic, and her creatinine level further inclined. Urgent hemodialysis was offered, and input-output was strictly monitored. A renal biopsy was performed.

On postsurgery day 3, she developed high-grade fever again, and therefore surgical site infection or femoral vascular catheter infection was suspected. Intravenous antibiotics were changed; intravenous flucloxacillin 500 mg 6-hourly was added according to the microbiology team’s opinion. The vascular catheter was removed, and catheter tip and blood samples, which were taken with aseptic nontouch technique, were sent for cultures. The culture results were negative for aerobic and anaerobic bacteria and fungi, and surgical site infection was also excluded. Findings of an echocardiogram ruled out infective endocarditis.

The patient’s full blood count showed hemoglobin of 7.6 g/dl, platelet count of 68 × 10^9^/L, and white cell count of 19.5 × 10^9^/L. Blood film revealed features of microangiopathic hemolytic anemia. The patient’s serum creatinine was 312 μmol/L. Her liver enzymes were within the normal range. Her coagulation profile was normal, including the thromboelastogram. Her lactate dehydrogenase (LDH) level was 3124 U/L. Her reticulocyte count was 7.27%. Her D-dimer was negative at 0.78 mg/L (< 1). Her Coombs test result was negative. With the blood film evidence and other test result findings, together with unexplained fever, TTP was suspected. She was admitted to the intensive care unit (ICU) and was initiated on therapeutic plasma exchange (TPE) together with cryo-poor plasma as the replacement fluid.

Her ICU stay was complicated with pulmonary hemorrhage with lower respiratory tract infection followed by respiratory failure requiring mechanical ventilation, and intravenous antibiotics were upgraded to meropenem 1 g 12-hourly and levofloxacin 500 mg once per day. Initially, plasma exchange was carried out daily (for 14 days) and then every other day (for 28 days). She was offered hemodialysis every other day for 14 days, every third day for 21 days, and every fourth day for 24 days. Hemodialysis was stopped following the improvement of urine output.

Histological examination of renal biopsy revealed fibrinoid necrosis of small arteries. The patient’s complement C3 was 57.6 mg/dl (normal range 90–180), and her complement C4 was 17.4 mg/dl (10–40). Results of tests for antineutrophil cytoplasmic antibodies, antinuclear antibody, and double-stranded deoxyribonucleic acid (DNA) were negative. Test results for hepatitis B surface antigen, hepatitis C antibody, human immunodeficiency virus, and VDRL (Venereal Disease Research Laboratory) were negative. Blood culture results were negative for *E. coli*. Investigation findings together with hypertension, blood picture features of microangiopathic hemolytic anemia, kidney injury, and pulmonary hemorrhage associated with respiratory failure prompted the diagnosis of atypical hemolytic uremic syndrome. ADAMTS-13 level and genetic studies were not done, owing to the unavailability of resources.

As the long-term management plan, the patient was offered long-term plasma exchange in a tapering regimen. Initially, two cycles per week were arranged. She gradually responded to treatment, and investigation results were improving. She stayed in the ICU for 14 days and was discharged from the hospital after a total of 69 days with two antihypertensive agents and a tapering regimen of oral prednisolone.

She was regularly followed up in the nephrology clinic, and TPE was offered once per week for 6 months and then tapered to once in 2 weeks, 4 weeks, 6 weeks, and 8 weeks. Following a total TPE course of 30 months, plasma exchange therapy was omitted. Her hemoglobin, platelet count, serum creatinine, LDH, reticulocyte count, and blood film were all within normal ranges.

## Discussion

Our patient represents a typical case of aHUS with a severe form of this disease. She developed a number of complications, such as septicemia, refractory hypertension, and pulmonary hemorrhage with respiratory failure. She was also in an oligoanuric state for almost 2 months. This case proves that early diagnosis and prompt treatment with TPE and renal replacement therapy will lead to a complete recovery from aHUS.

aHUS occurs due to complement-related abnormalities. The complement system is a part of the immune system that destroys microorganisms and damaged cells by enhancing the ability of antibodies and phagocytic cells via complement activation. It also promotes inflammation and attacks the pathogen’s cell membrane. It is activated via three pathways: the classical pathway, the alternative pathway (AP), and the lectin pathway. Uncontrolled activation of complements in AP is strongly associated with aHUS [4, 8, 9].

AP is continuously activated at a low level due to spontaneous C3 hydrolysis because it does not need specific initiators. C3 internal thioester bond is hydrolyzed by H_2_O easily and forms C3(H_2_O). Then it interacts with complement factor B (CFB) followed by cleavage by complement factor D (CFD) and generates C3(H_2_O)Bb. For the cleavage of C3 into C3a and C3b by C3 convertase enzyme complex, the C3(H_2_O)Bb molecule works as an initiator. However, complement factor I (CFI), complement factor H (CFH), and membrane cofactor protein (MCP) rapidly inactivate C3b. C3b may bind with CFB to form C3bB. This complex then is cleaved by CFD into Ba and Bb. Bb remains bound to C3b to produce C3bBb, which is the C3 convertase in AP. C3 convertase may recruit another C3b and produce C3bBbC3b, which is the C5 convertase. C5 convertase cleaves C5 into C5a and C5b molecules. The C5b molecule then binds to C6, C7, C8, and C9 to generate the membrane attack complex (MAC). MAC destroys the target directly by the formation of membrane pores [4, 10, 11].

Abnormalities of complement activation factors, such as CFB and CFD, and complement regulatory factors, such as CFI, CFH, and MCP, are associated with the development of aHUS. These abnormalities are due to uncontrolled complement activation by the loss-of-function variants in complement-regulatory proteins or gain-of-function variants in complement activation factors [4, 12]. Multiple genetic and acquired complement abnormalities have been identified.

CFH abnormalities have the highest frequency, accounting for 20–30% of cases. The main effects are impaired CFH binding to C3b and/or glycosaminoglycans on host cell surface and decreased cofactor activity [13–15]. Effects of CFI abnormalities are impaired CFI secretion and reduced proteolytic activity. MCP abnormalities have a frequency of 8–10% with reduction in MCP expression and decreased C3b binding and cofactor activity [16, 17]. Anti-CFH antibody inhibits the complement-regulatory function of CFH [10, 18].

C3 defects result in resistance to CFI-mediated inactivation of C3b and also generation of hyperactive C3 convertase [19]. Abnormalities in CFB lead to the resistance of convertase to decay by CFH and formation of hyperactive C3 convertase [20]. Several coagulation-related factor abnormalities are also recognized; thrombomodulin, diacylglycerol kinase epsilon, and plasminogen abnormalities are related to the development of aHUS [10, 21–23].

Clinical manifestations and outcome differ with underlying genetic or acquired defects in the complement system. Patients with aHUS, who have genetic mutations, are commonly affected during childhood (67%) [24]. Acute episodes of aHUS present with severe Coombs-negative hemolytic anemia, thrombocytopenia, and acute kidney injury. Extrarenal manifestations occur in 20% of cases [10, 25]. Among patients with CFH, CFI, and C3 mutations, 60–70% will progress to permanent renal damage or die during the initial episode or progress to end-stage renal failure (ESRF) following relapses. But in children with CFH antibodies, only 30% of patients end up with ESRF [24]. CFB abnormalities are related to worse renal prognosis, whereas CFH mutations are associated with more cardiovascular complications and a higher mortality [20, 25]. Ten-year survival in patients with CFH abnormalities is 50%, but patients with CFI or C3 mutations or anti-CFH antibodies have higher survival rates of 80–90% at 10 years [24]. The best prognosis is seen in patients with MCP mutations because they have complete remission at a rate of 80–90%. Even though recurrence is frequent, 80% of patients will not require hemodialysis [24, 25].

Current therapeutic options for aHUS are plasma exchange and eculizumab. Empirically, TPE is considered as the first-line treatment. It assists in clearing abnormal complement-related factors such as anti-CFH autoantibodies and provides complement-regulatory factors to the circulation [26]. Although TPE is associated with a reduction of mortality from 50% to 25%, in a 3-year follow-up, 48% of pediatric patients and 67% of adult patients died or progressed to ESRF [15, 26, 27]. TPE combined with immunosuppressive drugs such as corticosteroids and azathioprine or mycophenolate mofetil and an anti-CD20 antibody (rituximab) has shown long-term dialysis-free survival in 60–70% of patients [10, 28].

Eculizumab has become the gold standard treatment for aHUS with a good efficacy and safety profile. It is a monoclonal antibody against C5. It blocks the cleavage of C5 to C5a and C5b. C3b binding to C6, C7, C8, and C9 is prevented, and formation of MAC is blocked [29]. Eculizumab is recommended as the first-line therapy for children because of their increased risk of developing catheter-related complications such as infections with regard to TPE [30]. The duration of treatment with eculizumab in aHUS is unknown. Original approval was for lifelong treatment, whereas emerging evidence suggests that aHUS may not require indefinite eculizumab treatment and cessation may help mitigate sequelae of therapy [31]. However, when eculizumab is unavailable in resource-poor settings such as Sri Lanka, TPE may be the first treatment of choice. In our patient, TPE was a successful treatment, because she completely recovered following long-term therapy.

## Conclusion

Diagnosing aHUS is a challenge for a variety of reasons. It is a rare disease entity; complement diagnostics are not reliable; 30–50% of patients do not have genetic or acquired mutations in the complement system; TMA occur in the context of complement-activating conditions; no specific test is available to confirm the diagnosis of aHUS; and aHUS is a diagnosis of exclusion. Early diagnosis and therapy improves outcome. aHUS should be considered in all patients with TMA.

## Abbreviations

ADAMTS-13: A disintegrin and metalloproteinase with thrombospondin type 1 motif, member 13
aHUS: Atypical hemolytic uremic syndrome
AP: Alternative pathway
CFB: Complement factor B
CFD: Complement factor D
CFH: Complement factor H
CFI: Complement factor I
ESRF: End-stage renal failure
HUS: Hemolytic uremic syndrome
ICU: Intensive care unit
LDH: Lactate dehydrogenase
MAC: Membrane attack complex
MCP: Membrane cofactor protein
STEC: Shiga toxin-producing *Escherichia coli*
TMA: Thrombotic microangiopathy
TPE: Therapeutic plasma exchange
TTP: Thrombotic thrombocytopenic purpura
VDRL: Venereal Disease Research Laboratory
vWF: von Willebrand factor
